# Prevalence of Vitamin D Deficiency and Its Association With Anthropometric and Hematological Parameters Among Infants at a Tertiary Care Center in Meerut, India: A Cross-Sectional Study

**DOI:** 10.7759/cureus.73661

**Published:** 2024-11-14

**Authors:** Uday K Mandal, Ajeet Kumar Yadav, Mayukh Mukherjee, Ram K Nema, Hemant S Thakur, Vikas Yadav, Yogesh D Sabde

**Affiliations:** 1 Environmental Health and Epidemiology, Indian Council of Medical Research (ICMR) - National Institute for Research in Environmental Health, Bhopal, IND; 2 Pediatrics, Super Speciality Cancer Institute, Lucknow, IND; 3 Pharmacology, Bankura Sammilani Medical College, Kolkata, IND; 4 Environmental Biotechnology, Genetics and Molecular Biology, Indian Council of Medical Research (ICMR) - National Institute for Research in Environmental Health, Bhopal, IND

**Keywords:** anthropometric indices, cross-sectional study, hematological parameters, infants, northern india, vitamin d deficiency

## Abstract

Introduction: Vitamin D is crucial in calcium homeostasis and bone metabolism, and its deficiency is widespread even in sun-rich regions like India. The present study sought to elucidate the prevalence of vitamin D deficiency in infants and its potential association with various anthropometric and hematological parameters.

Methods: The study was conducted in a tertiary care hospital in northern India (in 2013-14) and involved 77 nine-month-old infants. Data collection included demographic information, anthropometric measurements, and laboratory parameters like levels of serum vitamin D, intact parathyroid hormone (iPTH), calcium, phosphorus, alkaline phosphatase (ALP), hemoglobin (Hb), packed cell volume (PCV), total RBC count, and mean corpuscular volume (MCV). Vitamin D status was classified based on serum 25-hydroxyvitamin D [25(OH) D] levels.

Results: The prevalence of vitamin D deficiency was 42.9% (33 out of 77 infants). Infants with vitamin D deficiency had significantly lower mean body weight (8.26 ± 0.89 kg) as compared to vitamin D non-deficient infants (8.72 ± 0.99 kg) (p = 0.04). Hematological analysis revealed significantly lower Hb levels (9.59 ± 1.68 g/dL) in the vitamin D deficient group compared to the vitamin D non-deficient group (10.35 ± 1.44 g/dL) (p = 0.04). Other significant differences included lower PCV (p = 0.03) and MCV (p = 0.002) in the vitamin D-deficient group.

Conclusion: This study highlights the high prevalence of vitamin D deficiency (42.9%) among infants and its significant association (p-value less than 0.05) with reduced body weight and hematological indices like Hb, PCV, and MCV. These findings underscore the need for routine screening, nutritional interventions, and vitamin D supplementation to address vitamin D deficiency among infants. Further research involving diverse populations is required to validate these results, further guiding optimum public health strategies.

## Introduction

Vitamin D is pivotal in maintaining calcium balance within the body [1]. It is a fat-soluble secosteroid, primarily synthesized in human skin upon exposure to sunlight [2]. The prevalence of vitamin D deficiency across age groups, including infancy, is being more frequently reported globally, including in the Indian subcontinent [3,4]. Another nationwide survey in India documented a prevalence of 13.7% in the one- to four-year age group [4]. Such a high prevalence of vitamin D deficiency is unexpected in a tropical nation like India, where ample sunlight is available throughout the year. Infants can naturally synthesize all the required vitamin D through casual sun exposure [3]. So, it is evident that, despite the widespread availability of sunlight, it does not guarantee adequate vitamin D levels in children [5]. Several environmental, sociocultural, and behavioral factors contribute to vitamin D deficiency. Key factors attributed to vitamin D deficiency are limited sunlight exposure resulting from frequent use of sunscreen, wearing covering clothing, having darker skin, living in high latitudes, and residing in cloudy areas [6,7]. Furthermore, chronic illnesses and malnutrition significantly heighten the risk of vitamin D deficiency for various underlying reasons [8,9]. Vitamin D supplementation is necessary for infants and children who manifest clinical features due to vitamin D deficiency or rickets and when vitamin D levels are in the deficient range (even if asymptomatic) [3,10].

However, it is essential to note that vitamin D supplementation cannot be administered indefinitely. Therefore, addressing the underlying issues that hinder continuous vitamin D synthesis in the body is crucial. Hence, we planned this cross-sectional study in term newborn babies delivered in a tertiary care hospital, intending to study the prevalence of vitamin D deficiency at nine months and its correlation with anthropometric indices and different hematological parameters.

## Materials and methods

This study was conducted in a government tertiary care hospital, LLRM Medical College, Meerut, Uttar Pradesh, India. We conducted this cross-sectional study with full-term newborn babies born at or beyond 37th completed weeks of gestation in a tertiary care hospital in northern India through either a normal vaginal (NVD) or lower segment cesarean section (LSCS) and whose parents gave informed written consent for blood sampling were recruited as study participants. The exclusion criteria were the presence of any congenital malformation and a history of hospital admission due to any cause. We collected the data when the child arrived at the hospital for routine immunization at the age of nine months.

Parents of children fulfilling the inclusion criteria were contacted, and the study details were shared. A semi-structured demographic variable questionnaire was completed after obtaining written informed consent. The anthropometric measurements, such as weight, length, occipitofrontal circumference (OFC), and mid-upper arm circumference, were recorded. We collected two blood samples: one in an EDTA vial for hematological parameters such as Hb, PCV, RBC count, and MCV, and another in a vacutainer vial for biochemical parameters such as serum vitamin D, iPTH (intact parathyroid hormone), calcium, phosphorus, and alkaline phosphatase (ALP). We classified the vitamin D status of infants based on their serum 25(OH) D levels, defining deficiency as <12 ng/mL, insufficiency as between 12 and 20 ng/mL, and sufficiency as >20 ng/mL, as per the revised 2021 recommendations of the IAP (Indian Academy of Paediatrics) [11].

Data were collected in the years 2013 and 2014. The data were collected on paper questionnaires and entered into a Microsoft Excel worksheet (Microsoft Corp., Redmond, WA). Subsequently, the data were exported into IBM SPSS software version 25 (IBM Corp., Armonk, NY) for analysis. The categorical variables were presented in frequency tables, while numerical variables were expressed as mean and standard deviation. For analysis purposes, we combined the vitamin D insufficient and sufficient groups into a single category, termed the "vitamin D non-deficient group," and compared it with the vitamin D deficient group. This approach aligns with the method used in previous nationwide surveys in India, enhancing the comparability and interpretability of the results [4]. Then, the means of the predictive variables (clinical parameters, anthropometrical parameters, and laboratory parameters) across the two categories of the outcome variable (vitamin D deficient and vitamin D non-deficient) were compared using the Student's t-test. Statistical significance was considered as a p-value less than 0.05.

The study was started after obtaining ethical approval from the Institutional Ethical Committee (IEC), LLRM Medical College, Meerut, Uttar Pradesh. Informed, written consent was taken from one of the parents of eligible participants in the regional language (Hindi) before data collection. Participant's records were kept confidential and anonymous. Infants whose parents opted to withdraw consent during the study period continued receiving standard care as per the hospital practice. Deficiency found in any of the biochemical parameters of any participants was communicated to parents, and the patient was referred to the pediatric department's OPD (Outer Patients Department) for further management.

## Results

We contacted 96 parents to participate in the study. Of these, 17 infants were deemed ineligible, two parents denied consent for blood collection, and ultimately, we were able to collect data from 77 infants.

Table 1 shows the baseline characteristics of the study participants. The infants had a mean age of 9.08 months (SD = 0.1). The infants had a mean birth weight of 2839 g (SD = 229). Out of 77 infants, 34 (44.15%) were female and 43 (55.8%) were male. The mean age (in months) of weaning was 5.97 (SD = 1.1) months. Of 77 infants, 50 (64.93%) were exclusively breastfed until six months of age (Table 1).

**Table 1:** Baseline characteristics of study population

Variables	Descriptive estimates
Age in months (mean and SD)	9.08 (0.1)
Birth weight in grams (mean and SD)	2839 (229)
Weight at 9 months in grams (mean and SD)	8524 (967)
Participants who were exclusively breastfed (number and percent)	50 (64.93%)
Age of introduction of complementary feeding in months (mean and SD)	5.97 (1.1)
Female numbers (%)	34 (44.2%)
Prevalence of vitamin D deficiency (vitamin D level <12 ng/mL)	42.9% (95% CI: 31.8% to 54.0%)

The prevalence of vitamin D deficiency (vitamin D level <12 ng/mL) in the study population was 42.9% (33 out of 77 infants), and the remaining 44 (57.1%) infants were vitamin D non-deficient (>12 ng/mL), covering both the insufficient (N = 25, 32.5%) and sufficient groups (N = 19, 24.7%).

**Table 2:** Clinical, anthropometric, blood bone, and hematological profile of both (vitamin D non-deficient vs. vitamin D deficient) groups Hb: hemoglobin, PCV: packed cell volume, RBC: red blood cell, MCV: mean corpuscular volume. ^#^P-value was considered significant if it was less than 0.05.

Variables	Vitamin D non-deficient (N = 44)	Vitamin D deficient (N = 33)	t-Test score	p-Value^#^ (t-test)
	Mean (standard deviation)	Mean (standard deviation)		
Duration of breastfeeding (months)	7.0 (2.61)	6.5 (2.55)	0.853	0.4
Complementary feeding introduced at (months)	5.89 (1.04)	6.09 (1.26)	−0.78	0.44
Diarrhea episodes	1.93 (0.97)	2.39 (1.34)	−1.75	0.08
Acute respiratory infection episodes	1.52 (0.85)	1.82 (1.40)	−1.147	0.26
Birth weight (kg)	2.94 (0.72)	2.86 (0.25)	0.665	0.51
Weight (kg) at 9 months	8.72 (0.99)	8.26 (0.89)	2.127	0.04
Occipito-frontal circumference (cm) at 9 months	44.0 (1.17)	43.9 (1.17)	0.18	0.86
Length (cm) at 9 months	71.5 (2.37)	71.4 (1.89)	0.191	0.85
Mid-upper arm circumference (cm) at 9 months	13.6 (0.84)	13.6 (0.83)	0.043	0.97
Serum calcium (mmol/L)	1.15 (0.06)	1.05 (0.08)	6.259	<0.001
Serum phosphorus (mg/dl)	4.56 (0.20)	4.53 (0.25)	0.485	0.63
Serum alkaline phosphatase (IU/L)	123.71 (48.46)	267.67 (116.75)	−7.386	<0.001
Serum parathyroid hormone (pg/ml)	31.67 (6.69)	53.56 (16.12)	−8.13	<0.001
Hb (gm/dl)	10.35 (1.44)	9.59 (1.68)	2.132	0.04
PCV (%)	31.67 (3.89)	29.03 (6.34)	2.239	0.03
RBC count (millions/cumm)	3.90 (0.71)	4.86 (4.45)	−1.401	0.16
MCV (fL)	79.89 (6.64)	73.56 (10.45)	3.24	0.002

We compared vitamin D-deficient and non-deficient groups with different variables. We found that most of the variables, i.e., duration of breastfeeding (vitamin D non-deficient 7.02 months, vitamin D deficient 6.52 months; p-value: 0.40), birth weight (vitamin D non-deficient 2.94 kg, vitamin D deficient 2.86 kg; p-value: 0.51), age of weaning (vitamin D non-deficient 5.89 months, vitamin D deficient 6.09 months; p-value: 0.44), number of diarrhea episodes (vitamin D non-deficient 1.93, vitamin D deficient 2.39; p-value: 0.08) and number of acute respiratory infection episodes (vitamin D non-deficient 1.52, vitamin D deficient 1.82; p-value: 0.26) did not have any significant association with vitamin D level (Table 2).

When comparing anthropometric indices at nine months of age across different vitamin D levels, we observed that babies deficient in vitamin D exhibited a mean lower weight at 9 months (8.26 kg) compared to non-deficient babies (8.72 kg), and this difference was statistically significant (p-value: 0.04). While values of other anthropometric indices such as mean head circumference (vitamin D non-deficient 44.0 cm, vitamin D deficient 43.9 cm; p-value: 0.86), mean height (vitamin D non-deficient 71.5 cm, vitamin D deficient 71.4 cm; p-value: 0.85), and mean mid-arm circumference (vitamin D non-deficient 13.6 cm, vitamin D deficient 13.6 cm; p-value: 0.97), showed no difference among two groups (Table 2).

We compared hematological parameters like hemoglobin (vitamin D non-deficient 10.35 gm/dl, vitamin D deficient 9.59 gm/dl; p-value: 0.04), packed cell volume (PCV) (vitamin D non-deficient 31.67%, vitamin D deficient 29.03 %; p-value: 0.03), total RBC count (vitamin D non-deficient 3.90 million/cubic mm), vitamin D deficient 4.86 million/cubic mm; p-value: 0.16), and mean corpuscular volume (MCV) (vitamin D non-deficient 79.89 fL, vitamin D deficient 73.56 fL; p-value: 0.002) between vitamin D deficient and non-deficient group, and we found that all the parameters were higher in vitamin D non-deficient group (Table 2).

## Discussion

The findings of the present study suggest a high prevalence of vitamin D deficiency in children at the age of nine months. Vitamin D deficiency/insufficiency status is significantly associated with the nutrition level of the children, mainly with blood hemoglobin level and body weight.

For years, vitamin D has been acknowledged primarily for its crucial role in managing calcium, phosphorus, and bone metabolism [1,12]. However, its significance has recently broadened, garnering attention as a critical regulator of diverse biological functions such as immune response, cell growth, and cardiovascular health [13,14]. Vitamin D has been shown to stimulate erythroid precursor cells, and receptors for vitamin D have been identified in various non-renal tissues, including bone marrow [13,15]. Vitamin D and its derivatives are found throughout many body tissues, as are the receptors for its active form, calcitriol. Calcitriol is pivotal in immune regulation, where it helps suppress the production of proinflammatory cytokines by various immune cells, thus providing a mechanism to curb excessive inflammation [16]. Both in vivo and in vitro studies reveal that calcitriol can decrease cytokine production, thereby reducing the inflammatory milieu and the chances of anemia [17].

On the other hand, severe iron deficiency anemia in infants aged 1 to 12 months has been linked to decreased plasma vitamin D concentrations. This phenomenon is thought to occur due to diminished intestinal vitamin D absorption caused by iron deficiency [18]. However, a study by Katsumata et al. on rats revealed that iron-deficiency anemia can impact bone formation and resorption. This effect occurs through the alteration of synthesis and metabolism of iron-dependent enzymes, such as prolyl and lysyl hydroxylases involved in collagen synthesis, and renal 25-hydroxyvitamin D 1-hydroxylase, which affects the synthesis of abnormal collagen and reduces plasma concentration of 1,25-dihydroxycholecalciferol. Consequently, this leads to decreased bone mineral density [19].

In our research, we have analyzed a total of 77 infants at the age of nine months. We have found that the mean hemoglobin level in the vitamin D deficient group (9.59 ± 1.68) is significantly lower than the mean hemoglobin level in the vitamin D non-deficient group (10.35 ± 1.44). These findings align with other studies where it was observed that vitamin D deficiency is associated with anemia or blood hemoglobin level [17,20-22]. In a study by Jin et al. on 102 Korean infants aged 3-24 months, he found that two-thirds of the anemic infants had concurrent vitamin D deficiency, with an odds ratio of 4.115. He also found that vitamin D deficiency was more prevalent in the iron deficiency anemia group (P = 0.008) [17]. A similar finding was observed by Grindulis et al., in which they found a significant association between vitamin D deficiency, low hemoglobin, and low serum iron levels in 145 Asian-origin children at 22 months of age [20]. Various other research conducted in different geographical settings has demonstrated that low vitamin D levels are associated with higher rates of anemia, reinforcing the hypothesis that vitamin D plays a significant role in hematopoiesis and iron metabolism [21,22]. These results show that hemoglobin levels and hemoglobin-associated blood parameters should be measured in children with low serum vitamin D concentrations and vice versa.

On the contrary, a Chinese population-based study by Chang et al. [23] found no significant association between anemia and vitamin D levels. The possible reason they mentioned was that elevated blood lead levels may influence the blood hemoglobin level. Similar findings with no association between anemia and vitamin D level were also reported by Abdul-Razzak et al. [24], Kersey et al. [25], and Ozsoylu and Aytekin [26].

In this study, it was observed that babies with vitamin D deficiency exhibited a lower average body weight compared to those who were not deficient in vitamin D. Specifically, the average weight of the vitamin D-deficient infants was recorded at 8.26 kg with a standard deviation of 0.89 kg. In contrast, babies with vitamin D levels of 12 ng/ml or higher (indicating no deficiency) showed an average weight of 8.72 kg, with a standard deviation of 0.99 kg. These data suggest a potential correlation between vitamin D levels and infant body weight. We found no significant association between vitamin D levels and other growth metrics such as height, head circumference, and mid-arm circumference. These findings align with the study by Voortman et al., where they found a similar association between lower body weight and vitamin D deficiency status [27]. They found that underweight children had a higher risk of vitamin D deficiency than normal-weight children, whereas there were no significant differences for overweight or obese children.

This finding contrasts with earlier research, which reported higher rates of vitamin D deficiency among obese children. Previous studies have suggested a potential link between obesity and lower vitamin D levels, possibly due to factors such as the sequestration of the vitamin in fat tissue, reduced mobility, or lower dietary intake [28-30]. However, our study did not replicate these findings, indicating that the relationship between vitamin D status and body weight may vary depending on different population dynamics, different sociodemographic levels, or methodologies used in research. This discrepancy underscores the complexity of vitamin D's role in health and the need for further investigations to clarify its interactions with obesity/body weight in children. These findings also warrant further investigation into the role of vitamin D in early growth and development.

Iron deficiency is recognized to hinder the absorption of fat and vitamin A in the intestine, implying a potential impairment in vitamin D absorption as well [18]. Iron deficiency could play a role in the onset of vitamin D deficiency. So, it may be hypothesized that supplementation with iron, i.e., correction of other nutritional deficiencies, might enhance vitamin D absorption in the small intestine, thereby elevating the concentration of vitamin D in the bloodstream [18].

Our study was limited to fewer participants, all from a single city in North India, and it did not account for variations in sunlight exposure or seasonal effects on vitamin D levels. Therefore, further research involving a more geographically diverse population is essential to ensure the findings can be applied broadly.

## Conclusions

The findings of this study reveal a high prevalence of vitamin D deficiency (42.9%) among infants in Meerut City of northern India, with significant associations (p-value < 0.05) observed between low vitamin D levels and adverse anthropometric and hematological parameters. Infants with vitamin D deficiency exhibited lower body weight, hemoglobin levels, PCV, and MCV compared to their non-deficient counterparts. The study emphasizes the need for the detection of other signs of malnutrition along with vitamin D deficiency and intervention through nutritional supplementation and vitamin D fortification to prevent deficiencies and their associated health complications.

## References

[1] Fleet JC (2017). The role of vitamin D in the endocrinology controlling calcium homeostasis. Mol Cell Endocrinol.

[2] Specker BL, Valanis B, Hertzberg V, Edwards N, Tsang RC (1985). Sunshine exposure and serum 25-hydroxyvitamin D concentrations in exclusively breast-fed infants. J Pediatr.

[3] Ziegler EE, Hollis BW, Nelson SE, Jeter JM (2006). Vitamin D deficiency in breastfed infants in Iowa. Pediatrics.

[4] Rana G, Abraham RA, Sachdev HS (2023). Prevalence and correlates of vitamin D deficiency among children and adolescents from a nationally representative survey in India. Indian Pediatr.

[5] Al-Daghri NM, Yakout S, Sabico S (2022). Establishing the prevalence of osteomalacia in Arab adolescents using biochemical markers of bone health. Nutrients.

[6] Kift R, Berry JL, Vail A, Durkin MT, Rhodes LE, Webb AR (2013). Lifestyle factors including less cutaneous sun exposure contribute to starkly lower vitamin D levels in U.K. South Asians compared with the white population. Br J Dermatol.

[7] Uday S, Högler W (2018). Prevention of rickets and osteomalacia in the UK: political action overdue. Arch Dis Child.

[8] Joshi M, Uday S (2023). Vitamin D deficiency in chronic childhood disorders: importance of screening and prevention. Nutrients.

[9] Holick MF (2007). Vitamin D deficiency. N Engl J Med.

[10] Misra M, Pacaud D, Petryk A, Collett-Solberg PF, Kappy M (2008). Vitamin D deficiency in children and its management: review of current knowledge and recommendations. Pediatrics.

[11] Gupta P, Dabas A, Seth A (2022). Indian Academy of Pediatrics revised (2021) guidelines on prevention and treatment of vitamin D deficiency and rickets. Indian Pediatr.

[12] (2024). Current understanding of the molecular actions of vitamin D. https://journals.physiology.org/doi/full/10.1152/physrev.1998.78.4.1193.

[13] Bikle D (2009). Nonclassic actions of vitamin D. J Clin Endocrinol Metab.

[14] Reichel H, Koeffler HP, Norman AW (1989). The role of the vitamin D endocrine system in health and disease. N Engl J Med.

[15] Norman AW (2006). Minireview: vitamin D receptor: new assignments for an already busy receptor. Endocrinology.

[16] Blazsek I, Farabos C, Quittet P (1996). Bone marrow stromal cell defects and 1 alpha,25-dihydroxyvitamin D3 deficiency underlying human myeloid leukemias. Cancer Detect Prev.

[17] Jin HJ, Lee JH, Kim MK (2013). The prevalence of vitamin D deficiency in iron-deficient and normal children under the age of 24 months. Blood Res.

[18] Heldenberg D, Tenenbaum G, Weisman Y (1992). Effect of iron on serum 25-hydroxyvitamin D and 24,25-dihydroxyvitamin D concentrations. Am J Clin Nutr.

[19] Katsumata S, Katsumata-Tsuboi R, Uehara M, Suzuki K (2009). Severe iron deficiency decreases both bone formation and bone resorption in rats. J Nutr.

[20] Grindulis H, Scott PH, Belton NR, Wharton BA (1986). Combined deficiency of iron and vitamin D in Asian toddlers. Arch Dis Child.

[21] Yoon JH, Park CS, Seo JY, Choi YS, Ahn YM (2011). Clinical characteristics and prevalence of vitamin D insufficiency in children less than two years of age. Korean J Pediatr.

[22] Yoon JW, Kim SW, Yoo EG, Kim MK (2012). Prevalence and risk factors for vitamin D deficiency in children with iron deficiency anemia. Korean J Pediatr.

[23] Chang L, Liu X, Shi H, Dai W, Wang H, Jiang Y (2014). Association of 25-hydroxyvitamin D with Hb and lead in children: a Chinese population-based study. Public Health Nutr.

[24] Abdul-Razzak KK, Khoursheed AM, Altawalbeh SM, Obeidat BA, Ajlony MJ (2012). Hb level in relation to vitamin D status in healthy infants and toddlers. Public Health Nutr.

[25] Kersey M, Chi M, Cutts DB (2011). Anaemia, lead poisoning and vitamin D deficiency in low-income children: do current screening recommendations match the burden of illness?. Public Health Nutr.

[26] Özsoylu Ş, Aytekin MN (2011). Vitamin D deficiency and anemia. Ann Hematol.

[27] Voortman T, van den Hooven EH, Heijboer AC, Hofman A, Jaddoe VW, Franco OH (2015). Vitamin D deficiency in school-age children is associated with sociodemographic and lifestyle factors. J Nutr.

[28] Absoud M, Cummins C, Lim MJ, Wassmer E, Shaw N (2011). Prevalence and predictors of vitamin D insufficiency in children: a Great Britain population based study. PLoS One.

[29] Kumar J, Muntner P, Kaskel FJ, Hailpern SM, Melamed ML (2009). Prevalence and associations of 25-hydroxyvitamin D deficiency in US children: NHANES 2001-2004. Pediatrics.

[30] Sioen I, Mouratidou T, Kaufman JM (2012). Determinants of vitamin D status in young children: results from the Belgian arm of the IDEFICS (Identification and Prevention of Dietary- and Lifestyle-Induced Health Effects in Children and Infants) Study. Public Health Nutr.

